# Patterns in Psychiatric Emergency Care: a Three-Month Statistical Approach

**DOI:** 10.1192/j.eurpsy.2025.1451

**Published:** 2025-08-26

**Authors:** A. De La Cruz Davila, M. Navarro, K. Muñoz, A. Gómez

**Affiliations:** 1 Psychiatry Service, Hospital Álvaro Cunqueiro, Vigo, Spain

## Abstract

**Introduction:**

Psychiatric emergencies represent a significant challenge for healthcare systems due to their impact on patients, families and also healthcare professionals. These emergencies often arise in crisis situations, requiring immediate and appropriate intervention. Effective management not only involves stabilizing the patient but attending to psychosocial factors and continuity of treatment. In recent years, there has been an increase in the demand for psychiatric emergency services attributed to various factors; this underscores the need to analyze the patterns of these emergencies in order to optimize available resources and improve patient care.

**Objectives:**

The present study provides information regarding demographic and clinical characteristics of the patients treated. The main goal is to identify trends, risk factors, and opportunities for improving critical situations management as well as and the effectiveness of procedures implemented in primary and specialized care.

**Methods:**

Currently there is no standardized method for collecting data on urgent psychiatric care, thus depending on the specific methodology of each center. In our hospital, a written request from the General Emergency department (where patients are initially received and attended) is mandatory; without it, patients are not assessed. Therefore, there is a reliable computerized record of daily attendances, data that has been collected retrospectively on a weekly basis until the study period is completed (June, July, and August 2024).

The following items have been studied: time of request, age, sex, patient origin, reason for the request, prior follow-up, management in emergencies, and discharge referral. Telephone calls for specific consultations have not been included in the record or considered psychiatric care as such.

**Results:**

There is a wealth of cross-referenced data that can be obtained form the collected information. In our opinion, the most interesting ones are those regarding referral reasons and discharging plans according to sex as well as age groups.

**Image 1:**

**Figure:**
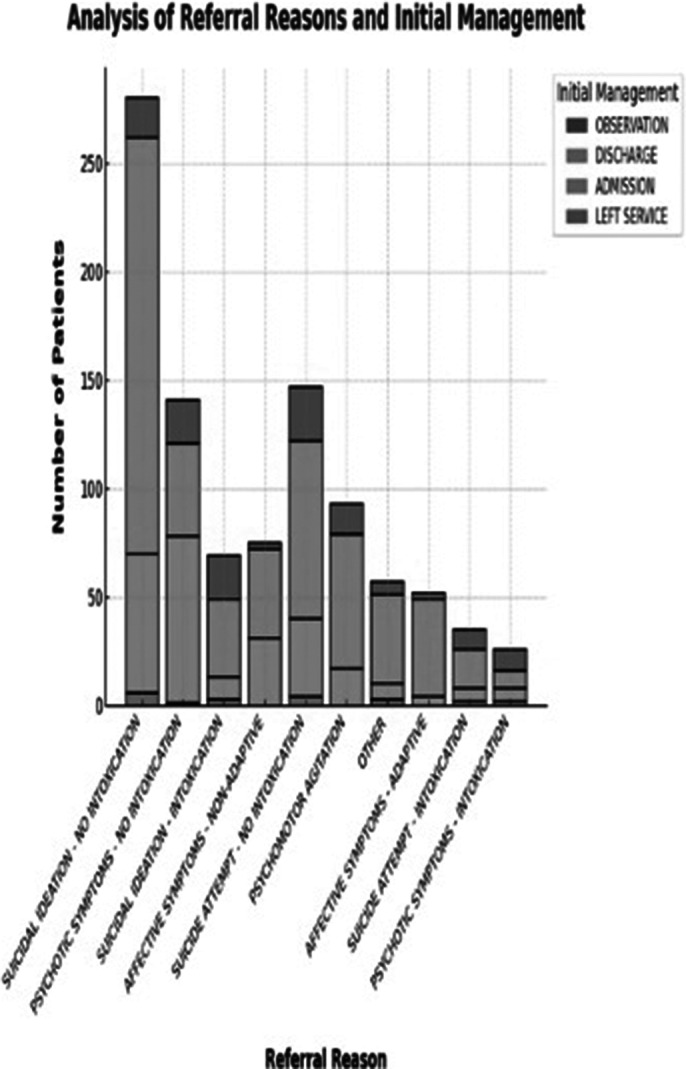

**Image 2:**

**Figure:**
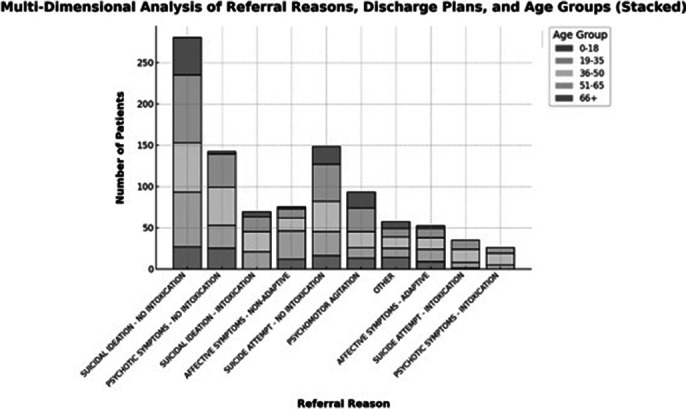

**Image 3:**

**Figure:**
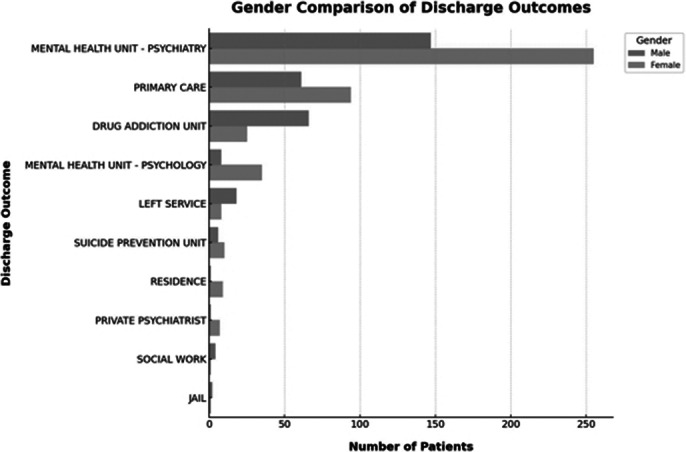

**Conclusions:**

The main conclusions are:

(1) More than 55% of patients present voluntarily, which could be interpreted as a positive indicator of mental health awareness, although it may also indicate a lack of prior support to prevent these crises.
(2) Of the patients assessed, 64.3% are discharged and 22.1% are admitted, with a low rate of service abandonment (1.8%). 64.3% of the total number had prior follow-up, suggesting effective ongoing care.
(3) More than 56% of the attendances received are women. It is essential to investigate whether this gender difference is due to a greater predisposition to seek help or differences in the incidence of psychiatric disorders.
(4) Regarding the reason of consultation, there is a high prevalence of suicidal ideation without prior intoxication (22.1%), reflecting the importance of preventive strategies in mental health and early crisis intervention.

**Disclosure of Interest:**

None Declared

